# Expression analysis of BACH1 with clinical variables using the US breast cancer patient cohort

**DOI:** 10.21203/rs.3.rs-3121163/v1

**Published:** 2023-07-05

**Authors:** N. Maritza Dowling, Galina Khramtsova, Olufunmilayo Olopade, Bok-Soon Lee, Jiyoung Lee

**Affiliations:** The George Washington University; The University of Chicago; The University of Chicago; The George Washington University; The George Washington University

**Keywords:** BACH1, MCT1, IHC score, Black women, breast cancer, basal-like subtype

## Abstract

**Background:**

Studies on functional roles of BACH1 reveal that BACH1 promotes cancer metastasis and regulates metabolic networks for metastatic processes. However, little is known about BACH1 protein expression in breast tumors and its relevance to clinical variables as a biomarker for patients with breast tumors.

**Methods:**

Using a tissue microarray (TMA) of breast tumor tissues isolated from a patient cohort (N = 130) expression of BACH1 and its target gene MCT1 (encoded by SLC16A1) were monitored by immunohistochemistry (IHC) assays and scored for further analyses. We examined the association between scores of BACH1 (Allredscoretotal) or MCT1 (Hscoretotal3×2×1x) with clinical variables including: breast cancer subtypes, tissue types, tumor size, patient’s racial/ethnic background, and age group. Groups were compared using the Mann–Whitney U test (or the non-parametric Kruskal–Wallis test when appropriate) for numerical data. A proportional odds ordinal logistic model was used to examine multiple covariates. Associations between variables were evaluated with the Spearman’s correlation coefficient.

**Results:**

BACH1 and MCT1 expression were detected in 90.76% (N = 118/130) and 92.30% (N = 120/130) of patients by IHC, respectively, in our study. After dichotomizing tumor size (small: 3–25 in diameter vs. big: 27–85 mm in diameter), BACH1 expression scores were significantly higher (*p* = 0.015) in the bigger tumor group (mean [SD]; 4.20 [1.796]) compared with the smaller tumor group (3.920 [1.693]). Of interest, we also observed significantly higher BACH1 scores (*p* = 0.004) in tumors from Black women (3.971 [1.514]; N = 69) compared with those of White women (3.02 [1.942]; N = 49). Consistent with mRNA expression analysis, BACH1 expression is most abundant in the basal-like tumors among all subtypes, specifically in Black women, whereas MCT1 expression scores are considerably higher in the basal-like tumors regardless of race. In addition, there was a positive association between BACH1 and MCT1 IHC scores in tumors from Black women, although a weak association between them in tumors from White women. In general, we did not detect associations between MCT1 IHC scores and race, tumor size, tissue types, or patient’s age.

**Conclusions:**

We found strong associations of BACH1 expression with tumor size and the basal-like subtype, respectively. Importantly, BACH1 expresses significantly higher in tumors from Black women than White women, as well as in the basal-like subtype of breast tumors from Black women. Our study suggests that BACH1 expression could serve as a potential race-associated biomarker indicating poor prognosis.

## Introduction

While overall survival rates and patient outcomes have improved, breast cancer causes the second most cancer-related death in women (1). Breast cancer-related death is mainly due to cancer metastasis resulting in secondary breast tumor formation in distant organs such as brain, liver, lung, and bone. The metastatic process consists of multiple steps, including epithelial to mesenchymal transition (EMT), migration and invasion, intravasation, extravasation and micro-metastasis (2). Additionally, tumor microenvironmental features, including hypoxia and tumor-infiltrating immune cell populations contribute to the cancer metastatic processes (3). Currently, targeted therapy blocking breast cancer metastasis is unavailable, although numerous molecules involved in the metastatic processes have been characterized.

A heme-binding transcription factor, BTB and CNC homology 1 (BACH1), has shown to promote breast cancer metastasis, particularly in triple-negative breast cancer (TNBC), the most aggressive and lethal subtype of breast cancer (4–7). Physiological functional roles of BACH1 include intracellular heme homeostasis, redox regulation, ferroptosis, and metabolic regulation in cancer cells (8–10). In breast cancer, relatively high levels of BACH1 cause increased migration and invasion, intravasation, and *in vivo* metastasis by upregulating transcriptional expression of matrix metalloproteinase 9 (MMP9) and C-X-C chemokine receptor type 4 (CXCR4) (11). Depletion of BACH1 expression in cancer cells was sufficient to reduce migration, invasion, intravasation, and metastasis of breast cancer. BACH1 also promotes EMT transition by downregulating epithelial cell markers through forkhead box 1 (FOXA1) inhibition in pancreatic cancer cells (12). In lung cancer, metastasis rates are increased by upregulated metabolic pathways such as hexokinase 2 (HK2) and glyceraldehyde-3-phosphate dehydrogenase (GAPDH) in a BACH1-dependent manner, that is further stabilized by an antioxidant-treatment or activated nuclear factor-erythroid factor 2-related factor (NRF2) (equivalent to mutation of kelch-like ECH associated protein 1 (Keap1)) (13, 14). In many tumor types including breast, BACH1 regulates gene expression to facilitate cancer cell migration, invasion, and metastasis at the transcription levels. Therefore, *BACH1* mRNA expression has shown to be associated with poor prognosis of breast cancer patients, in particular patients with basal-like TNBC tumors (6, 13, 14). Yet the expression levels of BACH1 proteins in breast tumors are understudied.

Previously we revealed that BACH1 regulates metabolic pathways, including lactate catabolism in TNBC cells, by suppressing monocarboxylate transporter 1 (MCT1 or encoded by *SLC16A1*) that facilitates pyruvate and lactate transport via a proton-dependent mechanism (15–17). MCT1 expression is highly associated with recurrent breast invasive ductal carcinoma and TNBC, as well as metastasis of melanoma (18–20). Mechanistically, MCT1 supports antioxidant regulation required for successful completion of the metastatic processes of melanoma cells. Inhibition of MCT1 mitigates aggressiveness of cancer cells. Targeting MCT1-dependent lactate flux contributes to improved outcomes for cancer patients, as MCT1 is associated with tumorigenesis. Currently, a small molecule targeting MCT1, AZD3965, is in a Phase I clinical trial as an anti-cancer therapeutic (21, 22), indicating MCT1 as a valuable biomarker for breast cancer.

In our study, we evaluated expression levels of BACH1 or MCT1 in breast tumor tissues to identify them as useful biomarkers for breast cancer metastasis and prognosis. Furthermore, expression of BACH1 or MCT1 was analyzed with clinical variables: breast cancer subtypes, tumor stages,

## Methods

### Patient tumor collection and tissue microarray construction

Archival formalin-fixed and paraffin-embedded (FFPE) breast tumor tissues were obtained from the Human Tissue Resource Center (HTRC) of the University of Chicago for tissue microarray (TMA) construction and were approved by the local Institutional Review Board (IRB # 10760B). The TMAs from FFPE *in situ*, invasive carcinomas tumor samples, and adjacent histological normal epithelium tissues served as internal positive controls. Tissue cores of 1 mm were arrayed into a new recipient paraffin block using an Automated Tissue Arrayer (ATA-27, Beecher Instruments, Sun Prairie, WI) as described previously (23). The location and identification of each tissue core were recorded in a Microsoft Excel database. Tumor tissues fixed using 10% formaldehyde, dehydrated using 70% ethanol, and embedded in the paraffin block were followed by cutting into sections of 3 *μ*m thickness before staining. Pathologic features, including diagnosis, grade, tumor size, and axillary lymph node metastasis were abstracted from pathology reports. The histology diagnosis, or grading of invasive breast cancer and carcinoma *in situ*, was performed separately by a pathologist (G.K) based on protocols of the College of American Pathologists and the World Health Organization (WHO) Classification Protocol for the examination of specimens from patients with invasive carcinoma of the breast (24). Breast cancer subtypes were defined as luminal A (estrogen receptor (ER)^+^ and/or progesterone receptor (PR)^+^, human epidermal growth receptor HER2^−^), luminal B (ER^+^ and/or PR^+^, HER2^+^), basal-like [(ER^−^, PR^−^, HER2^−^, CK5/6^+^) and/or epidermal growth factor receptor (EGFR)^+^], HER2^+^ (HER2^+^, ER^−^, PR^−^), or unclassified (negative for all five markers) as described previously (25).

### Immunohistochemistry

The TMAs contain a total of 130 patient tumors and were used for BACH1 and MCT1 staining using immunohistochemistry assays according to the standard protocol. The TMA slides were treated with antibodies against BACH1 (Santa Cruz, sc-271211) or MCT1 (Millipore Sigma, SAB2702323) in a 1:50 dilution in TE buffer (pH 9.5) overnight at 4 °C. The slides were counter-stained using Hematoxylin (Agilent, CS700) and scanned using HT. IHC staining was performed in Dr. Olopade’s lab and the HTRC at the University of Chicago.

### TMA scoring

The TMA slides stained for BACH1 and MCT1 were digitized on the 3D HISTECH panoramic whole slide scanner and analyzed. Scoring was based on intensity and percentage of positively stained cells; all discrepancies were resolved by a second examination using a digital slide image. The data corresponds to the results from the Human Protein Atlas (https://www.proteinatlas.org/). Scoring was confirmed using light microscopy and was performed independently and semi-quantitatively by one experienced pathologist (G.K) and one researcher (J.L). Tissues that failed for IHC staining and scoring were eliminated from the future analysis. For MCT1 staining, both intensity and percentage were scored for H-score calculation (26, 27). The intensity of protein expression was recorded as follows: 0 (no staining), 1 (weak staining, light brown), 2 (moderate staining, brown), or 3 (strong staining, dark brown). The proportion of tumor cells was scored as follows: 0 (< 10% positive cells), 1 (10–20% positive cells), 2 (21–50% positive cells) or 3 (> 50% positive cells).

For H-scores, the percentage of cells at each staining intensity level was calculated, and finally, an H-score was assigned using the following formula (28, 29):

[1×(%cells1+)+2×(%cells2+)+3×(%cells3+)]

The final score, ranging from 0 to 300, gives more relative weight to higher-intensity membrane staining in a given tumor sample. The sample can then be considered positive or negative on the basis of a specific discriminatory threshold.

#### NOTES

Originally, a score of less than 50 was considered negative (−) and scores of between 50 and 100 were considered weakly positive (+ 1). However, many centers now have lowered the threshold and only cases scoring less than 10 are considered negative, with those scoring between 10–100 being weakly positive.

For BACH1 staining, both intensity and proportion were scored using the Allred scoring system (30). The Allred score combines the percentage of positive cells and the intensity of the reaction product in most of the carcinoma. The two scores (intensity and proportion) are added together for a final score with 8 possible values. Scores of 0 and 2 are considered negative. Scores of 3–8 are considered positive. For analysis we convert one score to another to streamline the results (30, 31).

### Statistical analyses

For co-expression analyses using cancer patient data, we used non-parametric (distribution free) approaches. The Mann-Whitney U test (Wilcoxon rank-sum test) was used to compare outcomes between two independent groups. To compare three or more groups on a dependent variable we used the Kruskal–Wallis test. Post-hoc pairwise comparisons were adjusted using the Holm method to reduce type I error (32). Proportional odds ordinal logistic regression (PO-OLR) was used to control for multiple covariates. We used PO-OLR as a generalization of the Kruskal-Wallis test that extends to multiple covariates and interactions (33). Spearman’s rank-order correlation coefficient was employed to test the strength and magnitude of the relationship between ranked variables of interest. To assess the relationship between a dichotomous categorical variable and an ordinal variable we used the rank biserial correlation. Scatterplots and distribution plots were used to study the associations between the variables of interest in the sample and examine outliers and unusual observations. In particular, we estimated differences in BACH1 and the target gene MCT1 expression among multiple patient groups and clinical variables such as race (Black vs. White), age (below 55 vs. 55 and older), tumor subtypes (luminal A, luminal B, basal- like, and HER2-positive), tissue types (ductal carcinoma *in situ*, lobular carcinoma *in situ*, hyperplasia, lymph node metastasis, tumors), tumor size (diameter), tumor grades (grade 1: well differentiated, grade 2: moderately differentiated, grade 3: poorly differentiated, ND: not determined), and invasiveness (invasive vs. non-invasive). With the exception of post-hoc pairwise comparisons, all hypothesis tests were two-sided and carried out at an alpha level set at 0.05. Statistical analyses were conducted in *R* version 4.3.1 (R Core Team, 2023). The R package *rms*, Version 6.7–0 was used to fit the PO-OLR models (34).

## Results

### Detection of BACH1 and MCT1 in the breast tumor tissues using IHC analysis

**Figure 1:**
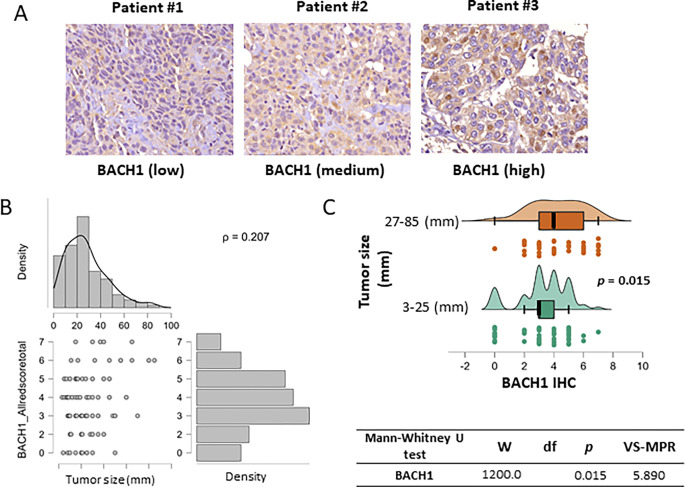
BACH1 expression is positively associated with breast tumor sizes (**A**) Representative images of BACH1 staining by IHC assays using breast tumor tissues. Staining scores from low to high are displayed. (**B**) BACH1 IHC score correlation with tumor size (diameter). Total sample N = 115, *Spearman’s* correlation coefficient = 0.207 (*p* = 0.027). (**C**) Patient tumors were separated in two groups based on tumor sizes (3–25 mm in diameter, N = 65) and (27–85 mm in diameter, N = 50). Distribution plot showing BACH1 IHC scores (Allredscoretotal) in two tumor size groups by Mann-Whitney U test and Vovk-SellkeMaximum *p*-Ratio (VS-MPR).

The transcriptional regulatory factor BACH1 activates or suppresses its target gene expression. As BACH1 mRNA levels predict breast cancer patient’s outcomes, BACH1 protein levels have potential as a biomarker to stratify cancer patients (6, 7, 10, 11, 13, 14). Thus we immediately asked whether BACH1 protein expression levels are accessible by IHC analysis using the breast tumor tissues. The TMA blocks contained tumor tissue samples collected from 130 patients with all subtypes (basal, luminal A, luminal B, HER2-positive) or tissue types (ductal carcinoma *in situ* breast cancer, lobular carcinoma *in situ*) at all stages, and normal tissues (Table 1). Available clinical information included patient’s race/ethnicity, age at diagnosis, tumor histology, tumor grade, tumor stage, and tumor size. Patients under age 55 at diagnosis comprised 40% (*N = 52/130*) of the total. The racial composition was 54.6% Black (*N = 71/130*), 40.7% White (*N = 53/130*), and 4.6% Asian or Hispanic (*N = 6/130*). The third group was excluded from the race-related analyses due to small sample size. The number of patients by descriptive measures are indicated in Table 1. For scoring of IHC staining, BACH1 IHC was evaluated by Allred scores total ranging from 0 to 7 and MCT1 IHC was scored by H score total3×2×1x ranging from 0 to 295. Median, mean, and standard deviation (SD) of IHC scores by clinical parameters are summarized in Table 2. Using IHC assays, we detected BACH1 expression in breast tumors collected and processed in our facility (Fig. 1A).

### BACH1 expression levels are positively associated with breast tumor size

Using BACH1 IHC scores we analyzed the association of BACH1 levels with biological variables of patient tumors. Given our relatively small sample size for subgroups of interest and non-normal distributions of BACH1 scores we used nonparametric models for the statistical analyses in our study (30). Our general hypothesis was to determine whether BACH1 levels were different by tumor characteristics. We tested BACH1 expression levels using IHC scores by tumor diameter size. Expression correlation analysis indicated that BACH1 IHC scores were positively correlated with tumor size. That is, BACH1 protein expression levels were higher in tumors with bigger diameter (*Spearman* coefficient = 0.207, *p* = 0.027) (Fig. 1B). Likewise, we further divided patient tumors into two groups based on tumor diameter such as small tumors (3–25 mm in diameter, *N* = 65) vs. big tumors (27–85 mm in diameter, *N* = 50) (**Supplementary Data Fig. 1**). The mean value of BACH1 IHC scores in smaller tumors was 3.292 (*SD* = 1.1693),whereas it was 4.2 (SD = 1.796) in bigger tumors. When BACH1 IHC scores were compared in the two tumor diameter groups using the Mann-Whitney U test for independent samples, we found a significant difference (*p* = 0.015) between the groups, showing the bigger tumor group having higher BACH1 IHC scores than the smaller tumor group (Fig. 1C). For additional statistical analysis, we used Vovk-Sellke Maximum *p* – Ratio (VS-MPR), based on a two-sided *p* -value, the maximum possible odds in favor of H over H equals 1/(−e *p* log(p )) for *p* ≤ .37 (33). The VS-MPR for BACH1 levels between small- and big-tumor groups was 5.890.

### BACH1 expression is different by tumor grades, not by tumor tissue types

**Figure 2:**
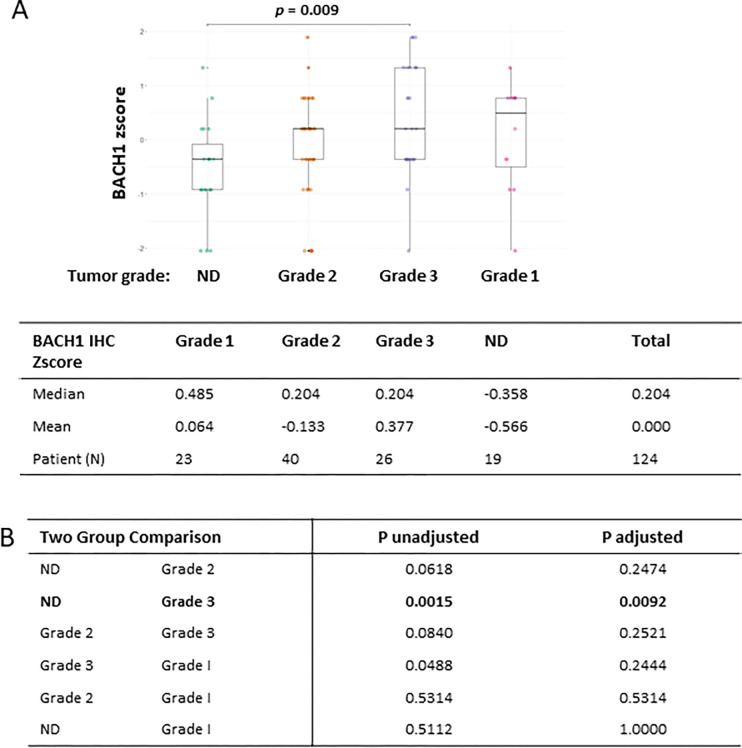
BACH1 expression is higher in the tumor grade 3 (**A**) Box plots indicating BACH1 IHC zscoresby breast cancer grades using the Kruskal-Wallis test. Median and mean of BACH1 IHC zscorein each groups, Grade 1 (N = 23), Grade 2 (N = 40), Grade 3 (N = 26), and Not Determined(N = 19), are shown. (**B**) Kruskal-Wallis multiple comparison with p-values adjusted with the Holm method are shown in the table.

We examined whether BACH1 levels were different by histological tumor grades as categorized by Grade 1: well differentiated, Grade 2: moderately and intermediate differentiated, Grade 3: poorly differentiated and dedifferentiated, and ND: cell type not determined. A Kruskal-Wallis rank sum test rejected our hypothesis that each tumor grade had the same BACH1 expression level by histological tumor grade. BACH1 levels were different in at least one grade (*p* = 0.015) (Fig. 2). Based on the post-hoc comparisons, BACH1 expression levels were significantly higher in the group of tumor grade 3, when compared to the group of ND (*p* = 0.009).

We also analyzed BACH1 IHC scores by tumor tissue type, and our data displayed similar levels of BACH1 by these tumor tissue types: ductal carcinoma *in situ* (DCIS), lobular carcinoma *in situ* (LCIS), hyperplasia, lymph node metastasis (LN-MTS), or tumors (T) (**Supplementary Data Fig. 2**). Metastases (MTS) and LCIS (luminal carcinoma *in situ*) were excluded in this comparison due to the limited sample size (*N* = 1 per group). Taken together, our data showed that BACH1 protein expression levels were more abundant in the group of tumor grade 3 defined as poorly differentiated or dedifferentiated in our patient cohort.

### BACH1 expression is higher in the basal-like breast tumor than the other subtypes

**Figure 3:**
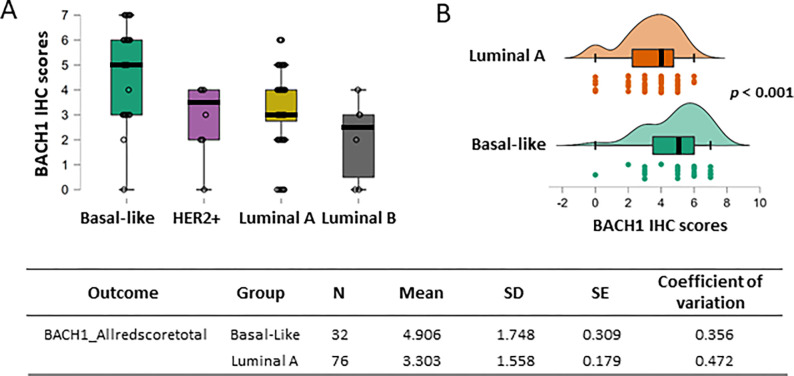
BACH1 expression is higher in basal-like subtypes than other breast tumor subtypes (**A**) Box plots indicating BACH1 IHC scores by breast cancer subtypes; basal-like (N = 32), HER2+ positive (N = 7), luminal A (N = 76), and luminal B (N = 5) subtypes. (**B**) Independent group comparison of BACH1 scores in basal-like (N = 32) and luminal A (N = 76) shows significantly higher BACH1 scores in basal-like tumors than the luminal A subtype. BACH1 IHC scores are significantly higher in the basal-like subtype than the luminal A subtype tumors (*p* < 0.001, Rank-Biserial Correlation = 0.517) by a Mann Whitney U-test for cross-comparison. Score mean, standard deviation (SD), standard error (SE) and coefficient of variation are shown by the subtype groups.

We further assessed whether BACH1 expression levels differed by tumor subtype. Prior research found that *BACH1* mRNA expression levels were highest in the basal-like subtype of breast tumors (6, 10). In our analyses, BACH1 protein levels assessed by IHC scores were also consistently higher in the basal-like subtype compared to the HER2-positive, luminal A, or luminal B subtypes (Fig. 3A). For statistical analysis, HER2 + and luminal B subtypes were excluded due to the limited sample size. The mean value of BACH1 scores was 4.906 (*SD* = 1.748; *N* = 32)in the basal-like tumors, while the mean was 3.303 (*SD* = 1.558; *N* = 76)in the luminal A tumors. Using a Mann Whitney U test for cross-comparison of basal-like and luminal A subtypes, BACH1 IHC scores were significantly higher in the basal-like tumor subtype (*p* < 0.001, Rank-Biserial Correlation = 0.517) (Fig. 3B).

Since BACH1 was higher in the basal-like tumor subtype, the most invasive subtype of breast cancer, we investigated whether BACH1 expression level was different by tumor invasiveness. Tumors were divided into two groups: invasive vs. non-invasive. The invasive group (*N* = 15) contained cancer types of MTS and LN_MTS; the non-invasive group (*N* = 109) contained cancer types of T, DICS, Fanconi anemia (FA), and hyperplasia. Unexpectedly, results revealed no significant differences in BACH1 levels between the invasive and non-invasive tumor groups using the Wilcoxon W test (**Supplementary Data Fig. 3A, B**).

#### BACH1 expression is higher in tumors from Black women than those from White women, particularly in the basal-like subtype

**Figure 4:**
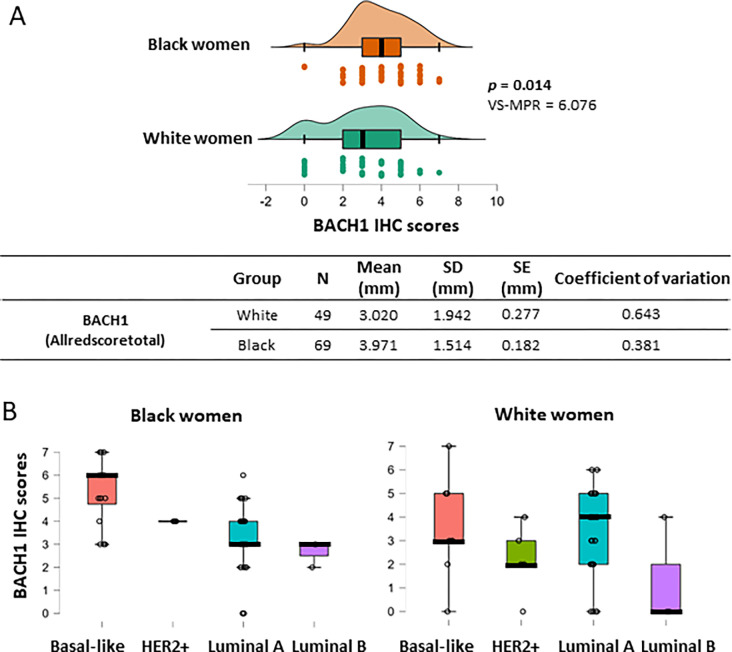
BACH1 expression score are higher in Black women than White women and in Basal-like subtype tumors from Black women (**A**) BACH1 IHC scores comparison in Black women (N = 69) and White women (N = 49) using the Mann-Whitney U test (*p* = 0.014, *VS-MPR is 6.076). Score mean of tumor size (millimeter, mm), standard deviation (SD), standard error (SE) and coefficient of variation are shown by ethnic groups. (**B**) Box plots indicate BACH1 IHC scores by tumor subtypes among Black or White patients.

In the United States, Black women have approximately 40% higher mortality rates from breast cancer than White women, although Black women have a lower incidence rate than White women (31). This cancer disparity requires immediate clinical and scientific attention to identify which patients are at higher risk and what factors contribute to disparity. Furthermore, precise and reliable biomarkers are needed to predict outcomes by patient’s biological traits including race. We analyzed BACH1 expression levels by race using the tumors from Black (*N* = 69) or White (*N* = 49) patients (Table 2). The mean value of BACH1 IHC scores from White women was 3.02 ± 1.942 (mean ± SD) and 3.971 ± 1.514 (mean ± SD) from Black women (Fig. 4A). Analyses using the Mann-Whitney U test indicated that tumors from Black women displayed significantly higher BACH1 scores than tumors from White women (*p* = 0.014, VS-MPR is 6.076). In addition, the total count of samples was higher for the tumors from Black women with higher BACH1 scores than tumors from White women (**Supplementary Data Fig. 4**). We further dissected subtypes of tumors among Black and White women to investigate whether BACH1 levels differed by tumor subtype and race. The mean value of BACH1 IHC scores was markedly higher in the basal-like subtype than other subtypes of tumors among Black women, whereas the mean of BACH1 scores was quite similar regardless of tumor subtype among White women (Fig. 4B). Because race is an important indicator for prognosis, we further investigated associations between BACH1 and race/ethnicity controlling for tumor grade or tissue type. We estimated a PO-OLR to evaluate whether race affected BACH1 expression while controlling for tissue type (invasive vs. non-invasive) or histological grades (tumor grade) (33). We found that tumors from Black women had significantly higher BACH1 expression levels compared to those from White women when tumor grades were used as a controlled variable (*p* = 0.0399). In addition, when tissue type (invasive or non-invasive) was used as a controlling variable, tumors from Black women expressed significantly higher BACH1 levels than those from White women (*p* = 0.014, VS-MPR is 6.076), indicating different levels of BACH1 by race despite tissue type. Taken together, BACH1 expresses significantly more in tumors from Black patients than from White patients, and particularly highest in the basal-like tumors from Black women regardless of tissue type or tumor grade.

### BACH1 expression has null correlation with patient’s age

Age is a known risk factor for many cancers including breast, and breast cancer incidence increases by patient age. Therefore we examined whether BACH1 expression levels in breast tumors correlate with patient’s age in our patient cohort. Patients in our analyses were classified into two age groups; a younger group (below 55 years old, *N* = 50) or an older group (above 55 years old, *N* = 73) and BACH1 IHC scores in both groups were compared using the Mann Whitney U test. The mean of BACH1 IHC scores of tumors from the younger age group was 3.78 (SD = 1.866) with coefficient variation (0.494), while the mean was 3.521 (SD = 1.725) with coefficient variation (0.490) from the older age group. BACH1 expression levels were not significantly different in the two groups (*p* = 0.257) (**Supplementary Data Fig. 5A**). We further separated patients into three age groups; age between 24–49 years old (*N* = 41), age between 50–65 years old (*N* = 39), age between 66–96 years old (*N* = 44) and compared BACH1 scores in multiple groups using the Kruskal-Wallis test. Statistical difference was not detected (*p* = 0.191) for BACH1 expression in three age groups, indicating that BACH1 expression levels are not significantly different by patient’s age (**Supplementary Data Fig. 5B**).

### MCT1 expression levels are higher in the basal-like tumors regardless of patient’s ethnicity

**Figure 5:**
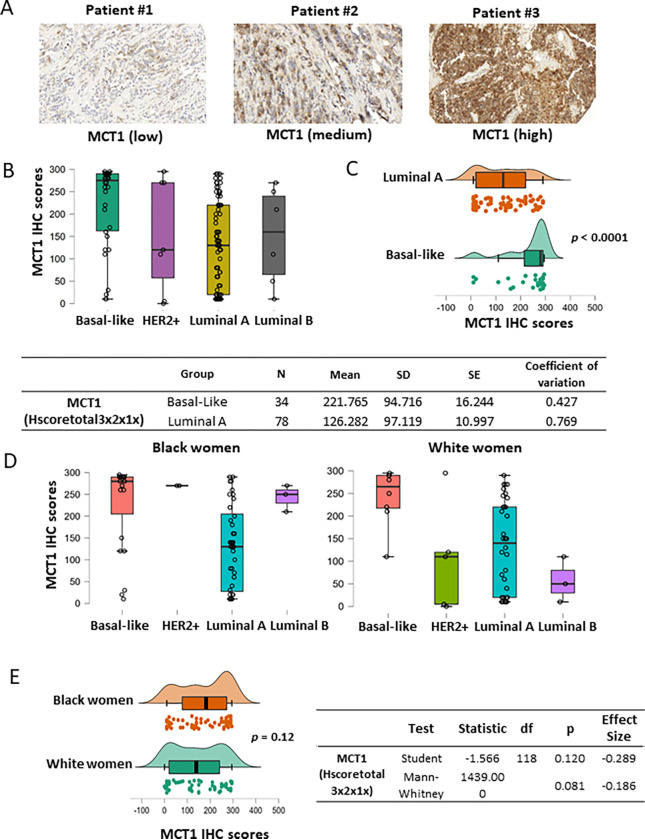
MCT1 IHC scores by breast cancer subtypes (**A**) Representative IHC images of MCT1 staining with low to high intensity in the breast TMA. (**B**) Boxplots showing MCT1 IHC scores by the subtypes of breast tumors (Basal-like N = 31, HER2+ N = 7, Luminal A N = 70, Luminal B N = 6). (**C**) MCT1 score analysis between luminal A (N = 78) vs. basal-like subtypes (N = 34) (*p*< 0.0001) by the Mann-Whitney *U*test. Effect size (0.561) is given by the Rank-Biserial correlation. (**D**) Boxplots showing MCT1 IHC scores by tumor subtypes from Black (left) or White women (right). (**E**) Distribution plots indicating that MCT1 scores have no statistical difference in between race groups by Student’s t test (*p*= 0.12) or Mann-Whitney U test (*p*= 0.081).

Upregulated MCT1 levels in breast cancer, especially in the basal-like subtype tumors, have previously been associated with poor outcomes of patients with breast cancer (18–20). We validated MTC1 expression by breast cancer subtypes using IHC assays in our patient cohort and analyzed its levels by race/ethnicity. Our TMA included MCT1 IHC staining scores (*N* = 120) from both Black women (*N* = 68) and White women (*N* = 52) (Table 2). The MCT1 IHC scores (Hscoretotal3×2×1x) ranged from 10 to 295 with mean 171.4 (SD = 105.9) for tumors from c tumors when compared with the HER2+, luminal A, or luminal B subtype tumors (Fig. 5B) (18–20). The two most abundant subtypes, basal-like (*N* = 34) and luminal A (*N* = 78), were compared for MCT1 expression levels. The mean value of MCT1 IHC scores for the basal-like tumors was 221.765 (SD = 94.716) and 126.282 (SD = 97.119) for the luminal A subtype, and their difference was significant according to the Mann-Whitney U test (*p* < 0.0001) (Fig. 5C). For the Mann-Whitney U test, effect size of 0.561 was given by the rank biserial correlation. Additionally, we investigated whether MCT1 expression showed racial disparity as BACH1 had shown (Fig. 4). When MCT1 expression levels were analyzed by tumor subtypes in each race/ethnicity, MCT1 scores were markedly different in the basal-like subtypes in both Black and White women (Fig. 5D). Since MCT1 expression showed enrichment in the basal-like subtypes in both races, we further asked whether MCT1 expression was equal or different by patient’s ethnicity/race. The analysis of MCT1 IHC scores indicates null difference of MCT1 levels between Black and White women, *p* = 0.081 by Mann-Whitney U test) (Fig. 5E). For the Mann-Whitney U test, effect size of −0.286 is given by the rank biserial correlation. Taken together, these demonstrate that MCT1 expression was substantially elevated in the basal-like subtype of breast tumors compared to other subtypes regardless of patient’s ethnicity.

### MCT1 expression is different in the tumor group of histological grade 3

**Figure 6:**
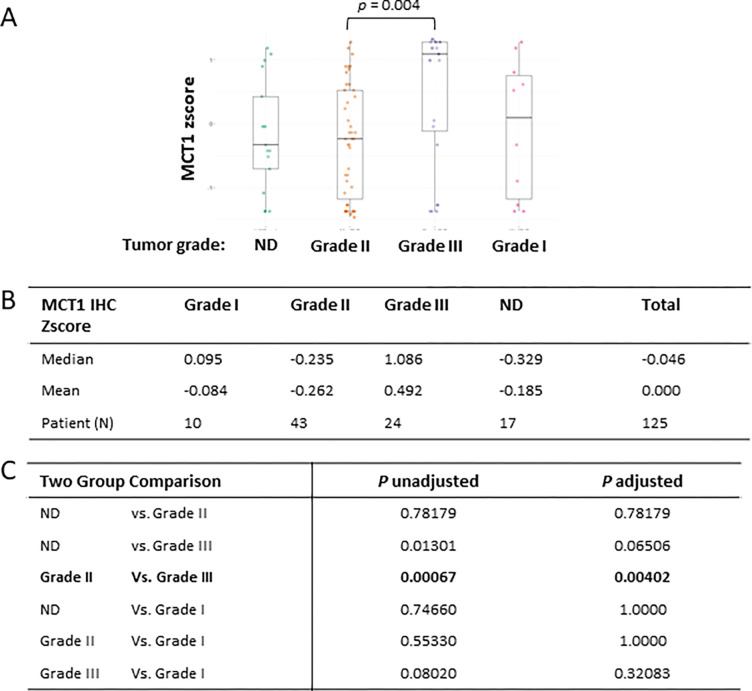
MCT1 expression is higher in the tumor grade 3 (**A**) Box plots indicating MCT1 IHC zscoresby breast cancer grades using the Kruskal-Wallis test. (**B**) Median and mean of MCT1 IHC zscorein each groups, Grade 1 (N = 10), Grade 2 (N = 43), Grade 3 (N = 24), and Not Determined (N = 17), are shown. (**C**) Kruskal-Wallis multiple comparison with *p*-values adjusted with the Holm method are shown in the table.

Next, we explored whether MCT1 levels were different or equal by histological tumor grades as we did for BACH1 analysis. We approached this question with the same hypothesis that each tumor grade had the same MCT1 expression levels, but the Kruskal-Wallis rank sum test result indicated that the MCT1 expression levels differed. Post-hoc comparisons indicated that MCT1 levels were significantly higher in the subgroup of tumor grade 3 than grade 2 (*p* = 0.004) in our patient cohort (Fig. 6).

### MCT1 expression has no association with tissue types, tumor size, or patient’s age

We further investigated if MCT1 expression differed by tumor tissue type and tumor size. MCT1 expression did not differ by either tumor tissue type (**Supplementary Fig. 6A**) or size (**Supplementary Fig. 6B**).

Since MCT1 is also abundant in the basal-like tumor that is the most invasive subtype of breast cancer, we questioned whether MCT1 expression levels differed by tumor invasiveness. Tumors were divided into invasive (*N* = 20) and non-invasive (*N* = 105) groups and compared with the Wilcoxon W test (**Supplementary Fig. 6C**). This analysis revealed no significant difference in MCT1 levels between the invasive and non-invasive tumor subgroups (*p* = 0.808).

Moreover, we assessed whether MCT1 expression levels differed by patient age. Comparisons of the MCT1 IHC scores of younger (below 55 years old, *N* = 52) and older (above 55 years old, *N* = 72) groups returned no significant differences (Mann-Whitney U test, *p* = 0.179) (**Supplementary Fig. 6D**). Likewise, comparing three age groups (24–49 years old, 50–65 years old, and 66–96 years old) found no statistical differences in the MCT1 IHC scores (Kruskal-Wallis test, *p* = 0.191). In summary, MCT1 expression levels were not associated with patient age, tumor size, tumor tissue types, or tumor invasiveness.

### Correlation between BACH1 and MCT1 expression in breast tumors

**Figure 7:**
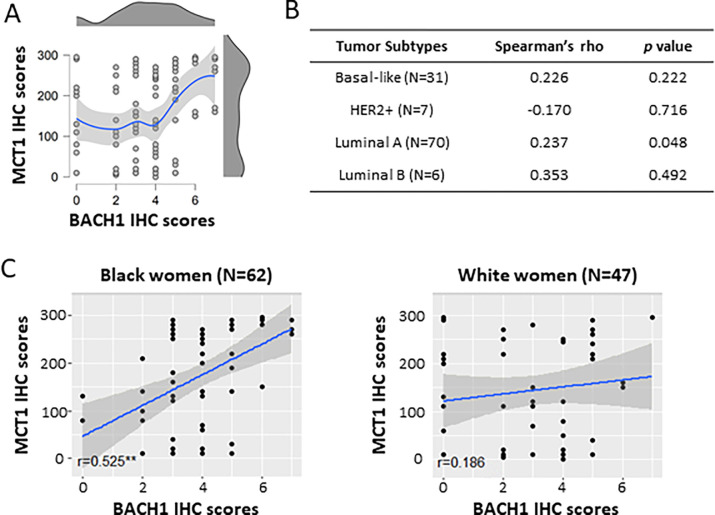
Expression analysis of MCT1 and BACH1 in breast tumors (**A**) Scattered plot and distribution plot of BACH1 and MCT1 scores show a positive expression correlation by Spearman Rank Correlation analysis (0.376, *p*< 0.001). The red dot in the distribution plot represents the median of the MCT1 variable at each level of BACH1. (**B**) Correlation analyses between BACH1 and MCT1 in tumor subtypes (patient numbers). *Spearman’s rho* and *p*-values are shown. (**C**) Scatter plot comparing the association of BACH1 and MCT1 by race, Black women (N=62, *Spearman’s Correlation* rho = 0.525, *p*= 0.00001, N = 62) vs. White women (*Spearman’s Correlation* rho = 0.186, *p*= 0.211, N = 47).

Our recent study revealed that BACH1 acts as a transcriptional suppressor of *SLC16A1*, which encodes MCT1 suppressing lactate catabolism in TNBC cells (17). We therefore investigated if BACH1 and MCT1 expression levels were correlated in our patient TMA cohort where we collected both BACH1 and MCT1 IHC scores. Using Spearman’s rank correlation analyses, we observed a positive correlation (0.376, *p* < 0.001) between BACH1 and MCT1 in total breast tumors (*N* = 114) (Fig. 7A). When we further analyzed their correlation in each tumor subtypes, we similarly observed a positive but insignificant trend between BACH1 and MCT1 in the basal-like (0.226, *p* = 0.222; *N* = 31), luminal A (0.237, *p* = 0.048; *N* = 70), and luminal B (0.353, *p* = 492; *N* = 6) subtypes (Fig. 7B). In contrast, we noticed an inverse, but not significant correlation between BACH1 and MCT1 IHC scores in the HER2 + subtypes (−0.17), from a smaller sample (*N* = 7). Interestingly, the expression correlation between BACH1 and MCT1 differed noticeably by patient’s race/ethnicity. Among Black patients, BACH1 and MCT1 displayed a strong positive correlation for their expression (0.525, *p* < 0.00001). In tumors among White women, however, there was a relatively weak and insignificant Spearman’s rank correlation for BACH1 and MCT1 expression (0.186, *p* = 0.211), indicating racial disparity between BACH1 and MCT correlation (Fig. 7C). Taken together, our data demonstrate a strong expression correlation between BACH1 and MCT1 in breast tumors from Black women, not from White women, and mostly from the luminal A breast tumor subtype.

## Discussion

The heme-regulatory transcriptional factor BACH1 promotes epithelial to mesenchymal transition (EMT), invasiveness and migration of cancer cells, and the metastatic process of breast tumors. BACH1 also regulates metabolic networks of cancer cells to support breast cancer metastasis. Therefore tumoral BACH1 expression predicts a worse outcome for patients with breast cancer. These data support our initiatives to investigate BACH1 protein levels in breast tumors from individual patients with various biological and racial background to identify any clinical relevance as an indicator.

One of the notable strengths of our study, nonetheless, is IHC staining for BACH1 in breast tumors. BACH1 has been noticed for its functional roles in tumorigenesis, and IHC assays have been used for many other solid tumors but not breast tumors. Here we showed BACH1 IHC staining in breast tumors with successful scoring for further analyses. Our BACH1 IHC staining along with complementary gene expression data from breast cancer patients would support BACH1 as a useful prognostic marker.

In the current study, we observed that BACH1 expression levels are positively correlated with tumor size. In addition, BACH1 expression levels are significantly higher in the tumors with histological grade 3. These suggest that BACH1 expression might be increased as tumors rapidly proliferate and/or BACH1 might advance its protein stability or transactivation in the tumor microenvironment by tumor extrinsic factors as tumors grow, which needs further investigation. Indeed, BACH1 mRNA expression is induced by hypoxia, which implies a tumor microenvironment effect (35). Oxygen tension or diffused oxygen concentration in the tumor microenvironment might directly or indirectly affect BACH1 protein expression levels in order to promote cancer metastasis (3).

A surprising finding in our study was that BACH1 expression in breast tumors differed by race. We identified that BACH1 expression is notably higher in breast tumors from Black women than those from White women. Particularly, BACH1 expression levels are higher in the basal-like subtype tumors than other subtypes from Black women. In contrast, BACH1 expression levels did not show statistical differences in the basal-like tumors or other types from White women. Results could be linked to differences in tumor sizes between the groups, Black women experience larger tumors (Table 2), and we found that BACH1 levels correlated with tumor size (Fig. 1). Our data suggested that BACH1 levels were particularly most abundant in the basal-like breast tumors from Black women, highlighting BACH1 as a race-related biomarker for patients. It also suggests further study to decipher genomic stability or amplification of BACH1 in tumors from Black women.

Akin to BACH1, MCT1 is a known prognosis marker for breast cancer (18, 19) and the inhibition of MCT1 using AZD3965 is currently under investigation in clinical trials to be applied as a targeted cancer therapy (22). It has previously been published that tumors expressed elevated MCT1 levels compared to adjacent normal tissue (19, 21, 22). In our current study, both Black and White women showed that MCT1 expression levels were significantly elevated in the basal-like tumors when compared with other subtypes, suggesting MCT1 as a useful biomarker for the patients with basal-like tumors to predict efficacy of AZD3965 treatment. Moreover, we identified that there is no appreciable difference or association of MCT1 expression with either tumor size, tumor tissue types, or patient age. Likewise, BACH1 showed no statistical differences among patients grouped by tissue type or patient age. These data strongly suggest that aging of breast cancer patients is less likely affecting the levels of BACH1 or MCT1 in tumors. Furthermore, other clinical variances including inflammation status of patients and metabolic disease status such as obesity or type II diabetes of patients might be of interest in future research on BACH1 or MCT1 expression.

Of note, there was a positive correlation between BACH1 and MCT1 expression using IHC scores, particularly among Black women. Since our previous study demonstrated that BACH1 suppresses MCT1-dependent lactate oxidation pathways in TNBC (17), a negative correlation was expected between BACH1 and MCT1 expression in the basal-like subtype of breast tumors. Unfortunately, small sample size would not provide enough power to compare the expression in basal-like or HER2 + subtypes of tumors. This could potentially be an experimental limitation of our current study, although the study included patient race, tumor subtypes, and clinical variables. We therefore recommend larger samples in future studies of expression among tumor subtypes. In addition, as we analyzed total IHC scores per tumor slide instead of cellular levels of each molecule’s expression, we suggest additional analyses of MCT1 and BACH1 levels at the single cell levels in each tumor types. This could reflect and advance study limitation of our current analyses, because correlation analyses using tumor tissues containing heterogeneous cancer cell populations could not access the possibility of exclusive expression between BACH1 and MCT1 per cell (36). Future studies using single cell images and individual IHC scores per cells would provide direct correlation between BACH1 and MCT1 expression in the TNBC or basal-like breast tumors.

## Conclusions

In our comprehensive analyses of BACH1 and MCT1 expression in breast tumors using IHC assays, we observed substantial increased expression levels of BACH1 in Black women, particularly in the basal-like breast tumors. BACH1 expression increased significant with tumor size, although MCT1 expression was not significantly correlated with tumor size. Higher tumor grade (grade 3) correlated with the highest levels of BACH1 and MCT1 in our patient cohort. A positive association between BACH1 and MCT1 was detected in breast tumors among Black women, whereas a null association was found in tumors among White women. Our data suggest that BACH1 might be a potential race-associated biomarker indicating poor prognosis of breast cancer.

## Figures and Tables

**Table 1:** Summary of demographic characteristics of patient tumor samples by race, age, and tumor size

Variables	Whites		Blacks	
**Age Groups (3 groups)**	N	%	N	%
24–49	17	30.9	21	28.0
50–65	22	40.0	24	32.0
66–96	16	29.1	30	40.0
Total	55		75	
**Age Groups (2 groups)**				
below 55	22	40.0	30	40.0
55 or older	33	60.0	45	60.0
Total	55		75	
**Tumor Size (diameter) Groups** ^a^				
3–25 mm	34	64.2	38	53.5
27–85 mm	19	35.8	33	46.5
Total	53		71	100.0

a No mass found or size not stated samples are excluded.

**Table 2:** Descriptive measures of tumors and IHC scores for BACH1 and MCT1

Race\Ethnicity		Tumor Size	Age at Diagnosis
White	Mean	24.96	57.85
	Median	24.00	58.00
	SD	14.560	14.904
	N	53	55
	Min	3	24
	Max	60	96
Black	Mean	29.69	61.01
	Median	25.00	61.00
	SD	19.084	16.467
	N	71	75
	Min	4	29
	Max	85	95
Total	Mean	27.67	59.68
	Median	25.00	61.00
	SD	17.390	15.842
	N	124	130
	Min	3	24
	Max	85	96

IHC scores	Race	N	Median	Mean	SD	Min	Max
MCT1_Hscoretotal3x2x1x ^a^	White	52	140	141.06	104.14	0	295
	Black	68	185	171.4	105.9	10	295
BACH1_Allredscoretotal ^a^	White	49	3	3.02	1.942	0	7
	Black	69	4	3.971	1.514	0	7

a Samples that failed IHC staining are excluded.

## Data Availability

All the data and materials including IHC images are available upon request

## Abbreviations

TNBC: triple-negative breast cancer
TCGA: the cancer genome atlas
BACH1: BTB and CNC Homology1
MCT1: monocarboxylate transporter 1
ER: estrogen receptor
PR: progesterone receptor
HER2: human epidermal growth factor receptor2
IHC: immunohistochemistry
TMA: tissue microarray
EMT: epithelial to mesenchymal transition
MMP9: matrix metalloproteinase 9
CXCR4: C-X-C chemokine receptor type 4
FOXA1: forkhead box 1
HK2: hexokinase 2
GAPDH: glyceraldehyde-3-phosphate dehydrogenase
NRF2: nuclear factor-erythroid factor 2-related factor
Keap1: kelch like ECH associated protein 1
